# A high mutation load of m.14597A>G in *MT-ND6* causes Leigh syndrome

**DOI:** 10.1038/s41598-021-90196-5

**Published:** 2021-05-27

**Authors:** Yoshihito Kishita, Kaori Ishikawa, Kazuto Nakada, Jun-Ichi Hayashi, Takuya Fushimi, Masaru Shimura, Masakazu Kohda, Akira Ohtake, Kei Murayama, Yasushi Okazaki

**Affiliations:** 1 Diagnostics and Therapeutics of Intractable Diseases, Intractable Disease Research Center, Juntendo University, Graduate School of Medicine, Tokyo, Japan; 2 Department of Life Science, Faculty of Science and Engineering, Kindai University, Osaka, Japan; 3 Faculty of Life and Environmental Sciences, University of Tsukuba, Tsukuba, Japan; 4 Life Science Center for Survival Dynamics, Tsukuba Advanced Research Alliance (TARA Center), University of Tsukuba, Ibaraki, Japan; 5 Center for Medical Genetics and Department of Metabolism, Chiba Children’s Hospital, Chiba, Japan; 6 Department of Pediatrics and Clinical Genomics, Faculty of Medicine, Saitama Medical University, Saitama, Japan; 7 Center for Intractable Diseases, Saitama Medical University Hospital, Saitama, Japan

**Keywords:** Mutation, Clinical genetics, Neurological disorders

## Abstract

Leigh syndrome (LS) is an early-onset progressive neurodegenerative disorder associated with mitochondrial deficiency. m.14597A>G (p.Ile26Thr) in the *MT-ND6* gene was reported to cause Leberʼs hereditary optic neuropathy (LHON) or dementia/dysarthria. In previous reports, less than 90% heteroplasmy was shown to result in adult-onset disease. Here, by whole mitochondrial sequencing, we identified m.14597A>G mutation of a patient with LS. PCR–RFLP analysis on fibroblasts from the patient revealed a high mutation load (> 90% heteroplasmy). We performed functional assays using cybrid cell models generated by fusing mtDNA-less rho0 HeLa cells with enucleated cells from patient fibroblasts carrying the m.14597A>G variant. Cybrid cell lines bearing the m.14597A>G variant exhibited severe effects on mitochondrial complex I activity. Additionally, impairment of cell proliferation, decreased ATP production and reduced oxygen consumption rate were observed in the cybrid cell lines bearing the m.14597A>G variant when the cells were metabolically stressed in medium containing galactose, indicating mitochondrial respiratory chain defects. These results suggest that a high mutation load of m.14597A>G leads to LS via a mitochondrial complex I defect, rather than LHON or dementia/dysarthria.

## Introduction

Leigh syndrome (LS, MIM 256,000) is a severe neurodegenerative disorder that is characterized by bilateral symmetric signs and symptoms of brainstem and/or basal ganglia disease^1,2^. The incidence of LS is estimated to be at least 1 in 40,000 live births. More than 85 disease-associated genes of LS encoded in the mitochondrial and nuclear genome have been reported^2,3^. Thirteen of the 37 mtDNA-encoded genes (MT-ND1, 2, 3, 4, 5, 6, MT-COIII, MT-ATP6, MT-TL1, MT-TK, MT-TV, MT-TW and MT-TI) have been found to be associated with LS. Our previous study of 104 Japanese LS patients described the clinical features, mitochondrial biochemical properties and genetic backgrounds in Japanese patients^4^. Most *MT-ND6* mutations cause Leber hereditary optic neuropathy (LHON), but some mutations have been reported to lead to LS. At present, two “Cfrm (confirmed)” mutations (m.14459G>A:p.Ala72Val and m.14487T>C:p.Met63Val)^5,6^ and three “Reported” mutations (m.14439G > A:p.Pro79Ser, m.14453G>A:p.Ala74Val and m.14600G>A:p.Pro25Leu)^7–9^ associated with LS have been registered in the MITOMAP database^10^. In our previous study, m.14439G>A:p.Pro79Ser was found to be associated with LS^7^, and a cybrid study revealed the pathogenicity of this variant. Recently, m.14597A>G has been shown to cause LHON^11^ and dementia/dysarthria based on information in ClinVar. m.14597A>G was associated with pathologies other than LHON or dementia/dysarthria, but it was unclear whether it was related to other disease types. We here report that a high mutation load of m.14597A>G causes LS with complex I deficiency.

## Results

**Figure 1﻿:**
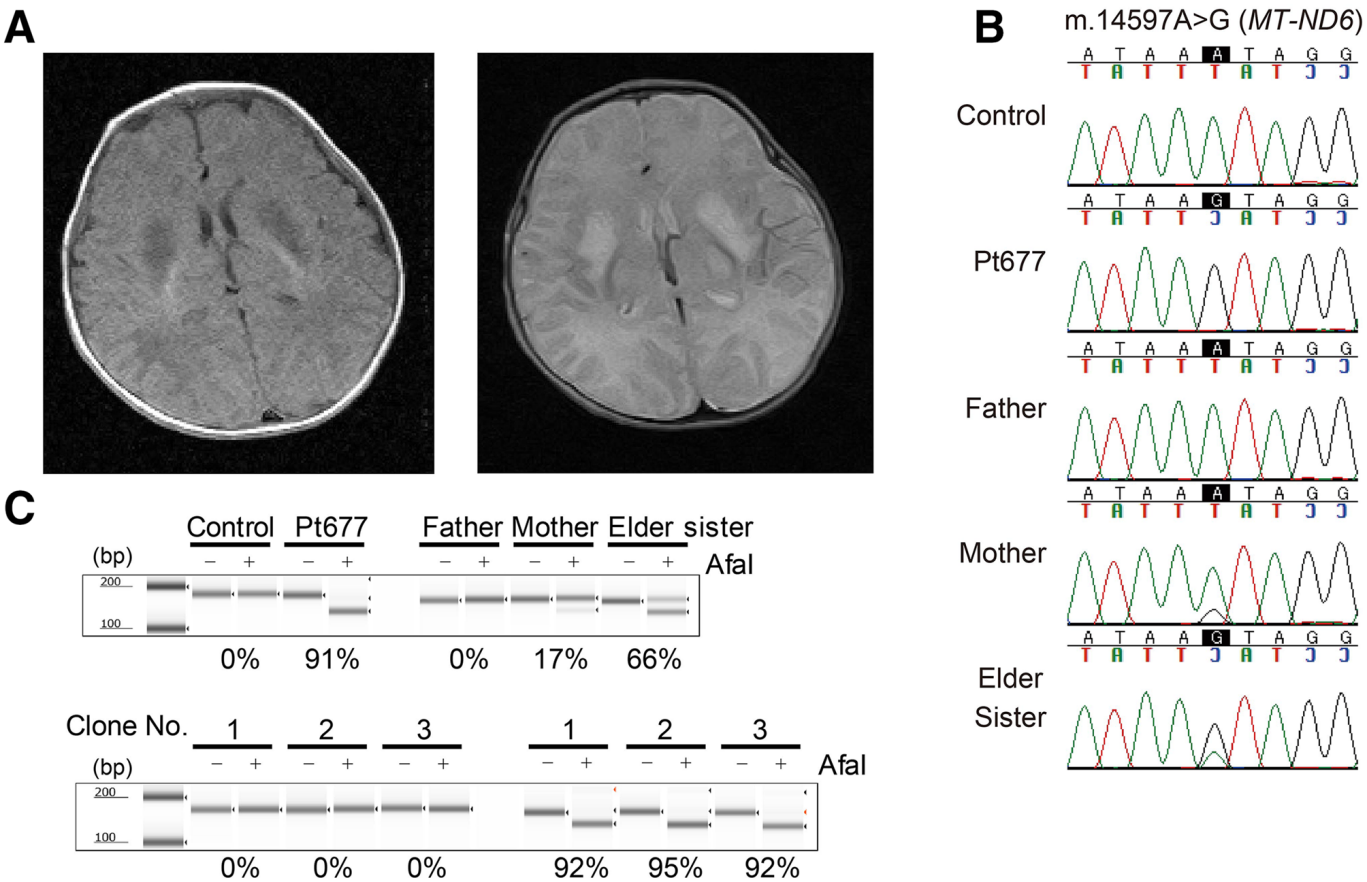
Clinical presentation and genetic diagnosis. (**A**) Magnetic resonance imaging at the age of 1 month and 15 days showed bilateral basal ganglia lesions with low signal on T1-weighted images (left) and high signal on T2-weighted (right) images. (**B**) Electropherograms of the m.14597A>G variant in the family. Sanger sequencing confirmed the maternal inheritance of the identified variant by the patient and his elder sister. (**C**) PCR–RFLP analysis of the m.14597A>G variant. Electrophoresis was performed using the TapeStation system (Agilent Technologies).

A Japanese boy (Pt677) was born at term, weighing 2434 g, as the second child to healthy parents with no consanguinity. His elder sister was healthy. There was no family history of mitochondrial disease including LS. No abnormalities were noted at the 1-month postnatal medical examination. Thereafter, the patient was found to exhibit lethargy, poor suckling, weight loss and myoclonic seizure and was admitted to hospital 1 month and 12 days after birth. On the fifth day of hospitalization, the patient had an apneic attack and was placed on mechanical ventilation. His general condition was stable with ventilator management, but spontaneous breathing was not observed, and continuous respiratory management with tracheostomy was required. Nissen fundoplication and gastrostomy were performed at the age of 10 months due to difficulty in oral intake. Magnetic resonance imaging showed an abnormal signal in the bilateral basal ganglia (Fig. 1A). Blood and cerebrospinal fluid (CSF) examinations repeatedly showed elevated lactate levels (blood lactate 2.5 mmol/L: reference range < 2.1 mmol/L, CSF lactate 4.4 mmol/L: reference range < 1.8 mmol/L). The patient was diagnosed with LS. His myoclonic seizure was controlled with phenobarbital and levetiracetam. Auditory brainstem resonance revealed hearing impairment in both ears. Optic nerve atrophy was identified at 2 years of age. The patient died of septic shock at the age of 6 years and 3 months, but autopsy was not carried out. We detected decreased activity of complex I in muscle tissue (M) and skin fibroblasts (F) (complex I, 0% [M] and 7.7% [F]; complex II, 159.3% [M] and 68.8% [F]; complex III, 208.2% [M] and 67.6% [F]; complex IV, 30.5% [M] and 18.9% [F] versus the respective control activity), whereas blue native-PAGE and western blotting showed normal mitochondrial complexes.

In a previous study, we did not identify any prioritized variant in nuclear-encoded genes or any confirmed pathogenic mutations in mitochondrially encoded genes from Pt677^12^. However, we found a mtDNA variant, m.14597A>G (p.Ile26Thr), in the *MT-ND6* gene which had not previously been reported to be associated with LS. Maternal inheritance was confirmed by Sanger sequencing of parental DNA (Fig. 1B). Sanger sequencing of the patient showed that the mutation was present at levels close to homoplasmy, and high heteroplasmy was observed in the elder sister, who had no symptoms. Furthermore, polymerase chain reaction-restriction fragment length polymorphism (PCR–RFLP) analysis revealed the m.14597A>G heteroplasmy levels of the patient and his family members (patient’s fibroblasts, 91%; mother’s blood 17%; elder sister’s blood, 66%) (Fig. 1C).

**Figure 2:**
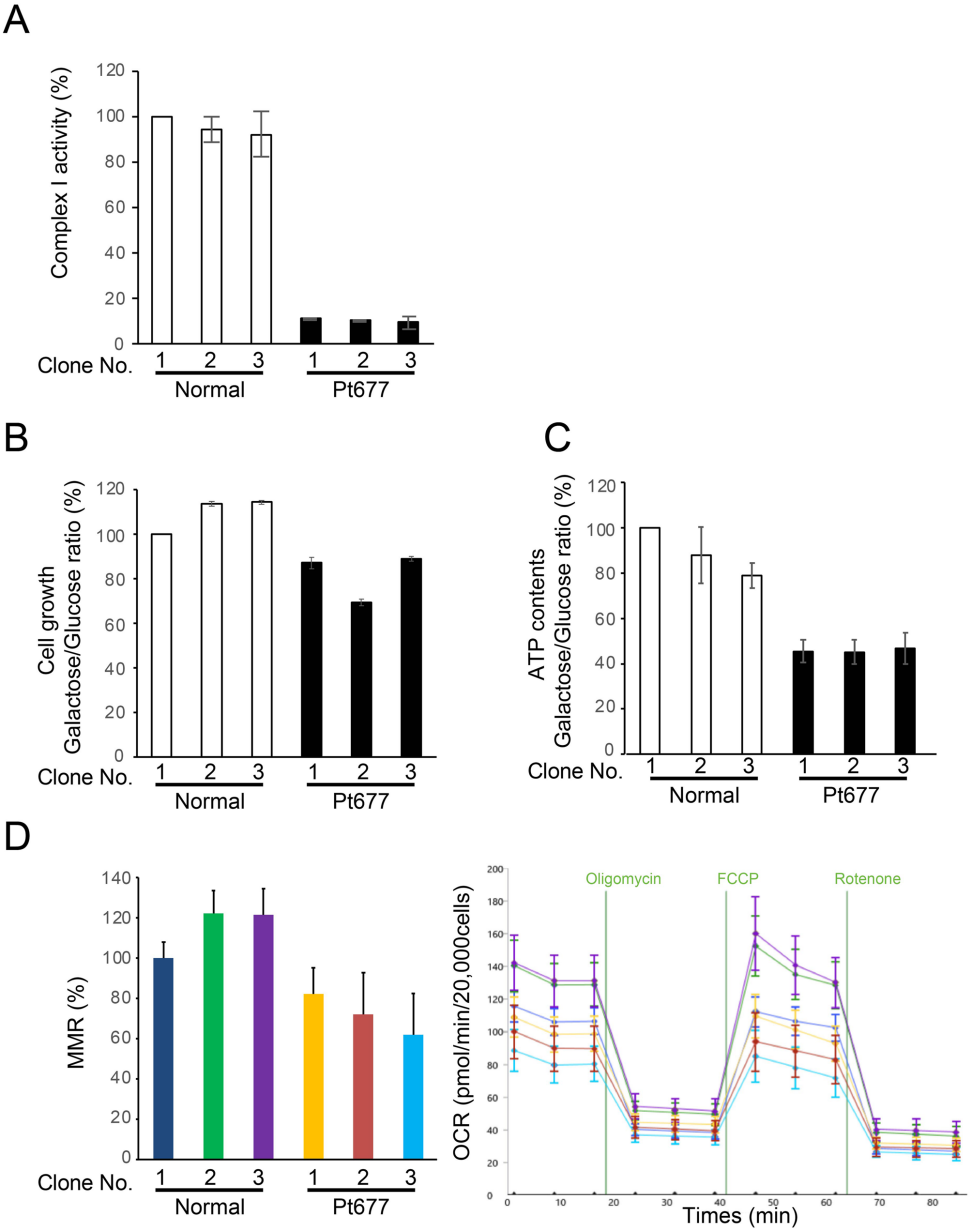
Phenotypic characterization of cybrid cell lines with the m.14597A>G variant. (**A**) Measurement of complex I activity in cybrid cell lines. Complex I (rotenone-sensitive NADH-ubiquinone oxidoreductase) activity was assessed spectrophotometrically. The values were normalized by CS activity. Error bars represent the standard deviation (n = 3). Cell growth and mitochondrial functions were compared in cells cultured in glucose or galactose (**B**,**C**). (**B**) Cell growth assay in cybrid cell lines. The ratio of values obtained in the galactose and glucose conditions was calculated. Error bars represent the standard deviation (n = 8). Experiments were repeated at least three times. (**C**) ATP measurement in cybrid cell lines. Data are shown as the mean ± SEM (n = 8). Experiments were repeated at least three times. (**D**) Oxygen consumption rate of cybrid cell lines from the patient compared with that of control cell lines 24 h after cell culture in medium containing galactose. OCR was monitored with the sequential addition of oligomycin (2 µM final), carbonyl cyanide 4-(trifluoromethoxy) phenylhydrazone (FCCP, 0.4 µM final), and rotenone (1 µM final) (right panel). The maximum respiration rate (MRR) corresponds to the OCR after the addition of FCCP minus rotenone-insensitive OCR (left panel). At least eight wells were analyzed for each sample. Error bars show the standard deviation. All values are calculated with normal clone 1 as 100%.

To further characterize the mitochondrial defects associated with m.14597A>G, we generated transmitochondrial cybrid cell lines derived from the patient’s fibroblasts carrying m.14597A>G using HeLa cells lacking mtDNA, as described previously^13^. Three cybrid clones derived from the patient’s fibroblasts had more than 90% heteroplasmy of m.14597A>G (Fig. 1C). The enzyme activity of complex I was significantly reduced in all three cybrid cell lines (Fig. 2A). Since cells grown in galactose rely mostly on oxidative phosphorylation (OXPHOS) instead of glycolysis to produce ATP, cells with an impaired OXPHOS system grow poorly in culture medium containing galactose. To test the galactose sensitivity of cybrid cells from the patient, the growth rate of cybrid cells was measured under galactose and glucose conditions. The cybrid cell lines derived from the patient showed a clear growth defect under the galactose conditions compared with control cells (Fig. 2B). We further examined the ATP content and oxygen consumption rate by culturing cells in media containing galactose. The ATP content of all patient cybrid cells was significantly reduced compared with that of control cybrid cells (Fig. 2C). We also detected a reduction of the maximum respiration rate in all patient cybrid cells compared with that of control cybrid cells (Fig. 2D). Together, these data suggest that the m.14597A>G variant is responsible for the defects in mitochondrial function.

## Discussion

Five *MT-ND6* mutations associated with LS have previously been reported as described above. This study identified the m.14597A>G variant in *MT-ND6* associated with LS. A low or median mutation load of m.14597A>G (blood: 25%, urine: 87%, fibroblasts: 76%) was previously shown to be associated with LHON^11^. In that previous report, the patient’s fibroblast cells with m.14597A>G showed reduced activity of electron transport chain and a high level of ROS production. The results are consistent with those shown in our cybrid cell experiments. In addition, the m.14597A>G variant was also reported to be associated with dementia/dysarthria based on the description in ClinVar. It was recorded that the identification of an association with dementia/dysarthria was based on a finding of de novo m.14597A>G with 49% heteroplasmy in a 31-year-old man with profound and progressive generalized dystonia, dysarthria and prominent perivascular spaces. These previously reported patients appeared to have moderate heteroplasmy and mild symptoms compared with our patient. The sister of our patient, who is currently asymptomatic, has 66% heteroplasmy in blood; she may thus have a similar disease course in the future. In conclusion, our findings suggest that more than 90% heteroplasmy would lead to the development of LS.

While the m.14597A>G variant causes p.Ile26Thr alteration, homoplasmic m.14596A>T, which results in p.Ile26Met alteration, was also reported to cause Leber optic atrophy and hereditary spastic dystonia^14^. In the ClinVar database, m.14598T>C (p.Ile26Val) is reported to be related to Parkinsonism and blindness. It is thought that p.Ile26 variants cause various diseases by producing different amino acid substitutions. p.Ile26 is located between two a-helices in the structure of the ND6 protein. p.Ile26Val causes a change from a branched-chain amino acid to a different branched-chain amino acid, which suggests that this substitution has a smaller effect than the other two substitutions. Moreover, p.Ile26Met involves a change between two nonpolar amino acids, while p.Ile26Thr involves a change from a nonpolar to a polar amino acid. The information of in silico prediction tools on MitImpact^15–17^ indicates that p.Ile26Val is less harmful than p.Ile26Thr and p.Ile26Met. “Damaging” or “deleterious” evaluations were given for p.Ile26Thr and p.Ile26Met by the same number of prediction tools, but the individual values were higher for p.Ile26Thr in most of the tools than those for p.Ile26Met, which suggests that p.Ile26Thr has a greater effect on the protein. In silico predictions suggest that the order of harmful effects is as follows: p.Ile26Thr > p.Ile26Met > p.Ile26Val. Thus, p.Ile26Thr is thought to cause the most severe form of disease, Leigh syndrome.

## Methods

This study was approved by the regional ethics committees of Juntendo University, Saitama Medical University, and Chiba Children’s Hospital and written informed consent was obtained from the patient’s parents. Specifically, consent was obtained for genetic and biochemical testing and publication o’s data. All methods were performed in accordance with relevant guidelines and regulations.

### Sequencing

Whole mitochondrial sequencing was performed as previously reported^12^. Long-range polymerase chain reaction was performed to purify DNA. Indexed paired-end mtDNA libraries were prepared with the Nextera XT DNA Sample Prep Kit and the Nextera XT Index Kit (Illumina) in accordance with the manufacturer’s guidelines. Sequencing was performed with 150-bp paired-end reads on MiSeq (Illumina).

The mtDNA variant was sequenced by Sanger sequencing. PCR products were directly sequenced using BigDye v3.1 Terminators and ABI 3130XL (Applied Biosystems).

### PCR–RFLP

The presence of the mtDNA variant was analyzed using PCR–RFLP. Mitochondrial DNA fragments including the *MT-ND6* gene were amplified with primers (the reverse primer contained an m.14600G>C mismatch). The amplified fragments (190 bp; m.14439–14628) were digested with the AfaI restriction enzyme, which can recognize the mutated DNA sequence. In the presence of m.15597G, the PCR product yields 160 bp and 30 bp DNA fragments. Fragment length and those molar concentrations were measured by the TapeStation system (Agilent Technologies).

### Cell culture and cybrid cell generation

Cells were cultured at 37 °C and 5% CO_2_ in Dulbecco's modified Eagle’s medium (DMEM with 4.5 g/l glucose; Nacalai Tesque) supplemented with 10% fetal bovine serum and 1% penicillin–streptomycin. To generate transmitochondrial cytoplasmic hybrids (cybrids), an mtDNA-less (rho0) derivative of HeLa cells was fused with the patient’s fibroblasts and normal human dermal fetal fibroblast as previously described^18^.

### Measurement of complex I activity

Mitochondrial respiratory chain complex I activities and citrate synthase activities were assessed as previously described^4^. Enzyme activity was measured using a Cary 300 UV-Vis spectrophotometer (Agilent Technologies) as per the manufacturer’s instructions. Protein concentration was determined by the bicinchoninic acid assay (Pierce™ BCA Protein Assay Kit, Thermo Fisher Scientific) and activity values were normalized by protein content. Finally, the complex I activity value was expressed as the percentage of citrate synthase activity.

### Measurement of oxygen consumption rate

The oxygen consumption rate was assessed as previously described^4^. Cybrid cells were seeded in a 96 well plate at 2 × 10^4^ cells/well with growth medium containing 25 mM glucose, and incubated for 24 h (37 °C, 5% CO_2_). After replacing the medium with unbuffered DMEM containing 1 mM sodium pyruvate, 2 mM glutamine, and 25 mM glucose or 10 mM galactose, the assay plates were incubated at 37 °C without CO_2_ for 1 h. Following the calibration of the sensor cartridge loaded with compounds including 2 μM oligomycin, 0.4 μM FCCP, and 1 μM rotenone, the experiments were started. The obtained data were normalized to the cell numbers determined using CyQUANT Cell Proliferation kit (Invitrogen).

### XTT cell proliferation assay

Cybrid cells were seeded in a 96 well plate at 1 × 10^3^ cells/well with growth medium containing 25 mM glucose, and incubated for 24 h. The number of cells was measured with an XTT Cell Proliferation Assay Kit (Cayman Chemical Company) 24 h after cell culture in medium containing 25 mM galactose or 25 mM glucose.

### Measurement of ATP

Cybrid cells were seeded in a 96 well plate at 1 × 10^3^ cells/well with growth medium containing 25 mM glucose, and incubated for 24 h. The ATP content was measured using an ATPlite luminescence assay kit (Perkin Elmer) 24 h after cell culture in medium containing 25 mM galactose or 25 mM glucose. Protein concentration was determined by the bicinchoninic acid assay (Pierce™ BCA Protein Assay Kit, Thermo Fisher Scientific). The values for ATP content were normalized to the mitochondrial protein concentration.

### Statistics

Data are expressed as the mean ± SEM. The statistical significance of differences was determined by two-tailed Student's t-test. A *p* value < 0.05 was considered significant.

### Ethics statement

This study was reviewed and approved by the regional ethics committees of Juntendo University, Saitama Medical University, and Chiba Children’s Hospital. Written informed consent was obtained from the patient’s parents.
